# Identification of Hub Genes Associated With Tuberculous Pleurisy by Integrated Bioinformatics Analysis

**DOI:** 10.3389/fgene.2021.730491

**Published:** 2021-12-03

**Authors:** Lei Shi, Zilu Wen, Hongwei Li, Yanzheng Song

**Affiliations:** ^1^ Department of Thoracic Surgery, Shanghai Public Health Clinical Center, Fudan University, Shanghai, China; ^2^ Department of Scientific Research, Shanghai Public Health Clinical Center, Fudan University, Shanghai, China; ^3^ TB Center, Shanghai Emerging and Re-emerging Infectious Diseases Institute, Shanghai, China

**Keywords:** tuberculous pleurisy, differential gene expression analysis, weighted gene co-expression network analysis, the differential co-expression genes, biomarkers

## Abstract

Improving the understanding of the molecular mechanism of tuberculous pleurisy is required to develop diagnosis and new therapy strategies of targeted genes. The purpose of this study is to identify important genes related to tuberculous pleurisy. In this study, the expression profile obtained by sequencing the surgically resected pleural tissue was used to explore the differentially co-expressed genes between tuberculous pleurisy tissue and normal tissue. 29 differentially co-expressed genes were screened by weighted gene co-expression network analysis (WGCNA) and differential gene expression analysis methods. According to the functional annotation analysis of R clusterProfiler software package, these genes are mainly enriched in nucleotide−sugar biosynthetic process (biological process), ficolin−1−rich granule lumen (cell component), and electron transfer activity (molecular function). In addition, in the protein-protein interaction (PPI) network, 20 hub genes of DEGs and WCGNA genes were identified using the CytoHubba plug-in of Cytoscape. In the end, RPL17 was identified as a gene that can be the biomarker of tuberculous pleurisy. At the same time, there are seven genes that may have relationship with the disease (UBA7, NDUFB8, UQCRFS1, JUNB, PSMC4, PHPT1, and MAPK11).

## Introduction

Tuberculosis is a very ancient disease, and some studies have pointed out that *Mycobacterium tuberculosis* infected humans five thousand years ago (Sinha et al., 2021). Tuberculous pleurisy is a kind of extrapulmonary tuberculosis, which also includes bone tuberculosis, lymphoid tuberculosis and so on (Reichler et al., 2020). According to the World Health Organization’s 2019 global report on tuberculosis, tuberculous pleurisy accounts for 30 per cent of all tuberculosis in developing countries (WHO Global, 2019; Tuberculosis ProgrammeGlobal, 2020). But at present, there is still no more accurate diagnostic means for tuberculous pleurisy, so it is impossible to achieve early diagnosis and early treatment of tuberculous pleurisy (Tuberculosis ProgrammeGlobal, 2020).

**FIGURE 1:**
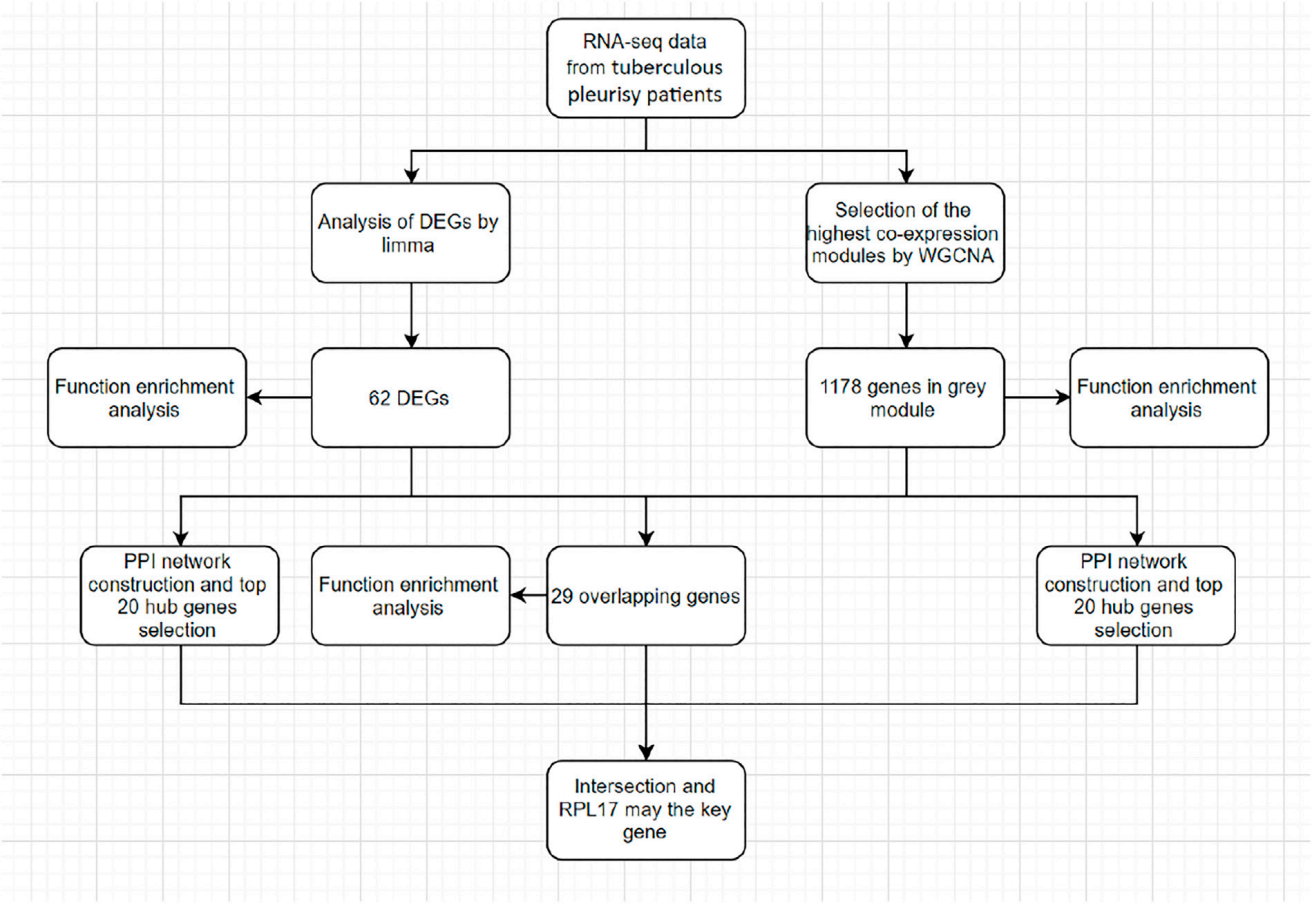
Study design and workflow of this study.

With the development of genome technology, bioinformatics is widely used in gene expression profile analysis (De Welzen et al., 2017). Figure 1 shows the research design and workflow of this study. Disease-specific biomarkers were found. Weighted gene co-expression network analysis of (WGCNA) is an important method to understand gene function and gene association from genome-wide expression (Langfelder and Horvath, 2008). WGCNA can be used to detect the co-expression modules of highly related genes and interested modules related to clinical characteristics (Zhang and Horvath, 2005), providing great insight for predicting the function of co-expression genes and finding genes that play a key role in human diseases (Li et al., 2018). In addition, another powerful analysis in transcriptomics is differential gene expression analysis, which provides a way to study the molecular mechanisms of genomic regulation and to find quantitative changes in expression levels between the experimental group and the control group (Saris et al., 2009; Yang et al., 2014; San Segundo-Val and Sanz-Lozano, 2016). This difference in gene expression may lead to the discovery of potential biomarkers for specific diseases. Therefore, two methods are used, combined with the results of WGCNA and differential gene expression analysis, to improve the recognition ability of highly related genes, which can be used as candidate biomarkers.

In this study, through the bioinformatics analysis of the sequencing data of pleural tissue collected during the operation, we hope to find the biomarkers that can be used for early diagnosis or treatment of tuberculous pleurisy. To contribute to the diagnosis and treatment of tuberculous pleurisy.

## Materials and Methods

The workflow for screening hub genes is shown in Figure 1. We describe the screening process in detail in each section.

### Acquisition of Transcriptome Sequencing Data

All the patients signed the informed consent form and completed the PET-CT examination before operation. During the operation of each patient, we obtained the pleura with high metabolic activity (PET-high) and low metabolic activity (PET-low) according to FDG PET-CT. Total RNA of each sample was extracted by TRIzol Reagent (Invitrogen, US) and RNeasy Mini Kit (Qiagen, Germany) according to the instructions of kit. Total RNA of each sample was quantified by Agilent 2100 Bioanalyzer (Agilent Technologies, United States), NanoDrop (Thermo Fisher Scientific Inc., United States). Total RNA (1 μg) with the value of RNA integrity number (RIN) > 7 was used for library preparation, which conducted by the NEBNext Ultra II Directional RNA Library Prep. Kit of Illumina. The rRNA was depleted from total RNA using Ribo-Zero™ rRNA removal Kit (Illumina, United States), and cDNA libraries were generated by protocols. Then, libraries with different indices were multiplexed and loaded on Illumina HiSeq instrument (Illumina, United States) for 2 × 150 paired-end sequencing in the Medical Laboratory of Nantong ZhongKe. Finally, the row reads were transformed as gene expression matrix by Trimmomatic (version 0.30), which was used to subsequently analysis. A total of 15 samples were included in the study, of which nine samples were PET-high and six samples were PET-low. This study was approved by the Ethics Committee of the Shanghai Public Health Clinical Center.

### Identification of Co-Expression Module Based on WGCNA

Co-expression network is a gene screening method which is based network that can be used to screen possible biomarkers and therapeutic targets. In this study, we constructed a co-expression network based on gene expression matrix and implemented it using R-package WGCNA (Langfelder and Horvath, 2008). WGCNA was used to explore the modules of highly correlated genes among samples for relating modules to external sample traits. To build a scale-free network, soft powers *β* = 3 and 20 were selected using the function pickSoftThreshold. Next, the adjacency matrix was created by the following formula:

aij=|Sij|β

aij: adjacency matrix between gene i and gene j

Sij: similarity matrix which is done by Pearson correlation of all gene pairsβ: softpower value,

and was transformed into a topological overlap matrix (TOM) as well as the corresponding dissimilarity (1-TOM). Afterwards, a hierarchical clustering dendrogram of the 1-TOM matrix was constructed to classify the similar gene expressions into different gene co-expression modules. To further identify functional modules in a co-expression network, the module-trait associations between modules, and clinical trait information were calculated according to the clinical data (Wang et al., 2019). Therefore, modules with high correlation coefficient were considered candidates relevant to clinical traits, and were selected for subsequent analysis.

### Identification of DEGs and Interaction With the Modules

We used R-package limma to screen DEGs, and DEGs were screened based on gene expression matrix (Ritchie et al., 2015). The limma package was used to analyze sequencing data and microarray data, and was a commonly used tool in bioinformatics. The screening criterion of DEGs were as follows: *p*-value < 0.05 and |logFC| > 1. The DEGs were visualized as a volcano plot and heatmap by using the R package ggplot2 and pheatmap. Subsequently, the overlapping genes between DEGs and co-expression genes that were extracted from the co-expression network were used to identify potential valueable genes, which were presented as a Venn diagram using the R package VennDiagram (Chen and Boutros, 2011).

### Functional Annotation for Genes

In order to describe the function and pathway of the selected gene, we conducted analysis using R package clusterProfiler (Yu et al., 2012), with a cut-off criterion of adjusted *p* < 0.05. Gene Ontology (GO) analysis consists of three different parts, biological process (BP), cellular component (CC), and molecular function (MF), which can accurately describe the function of the gene. Kyoto Encyclopedia of Genes and Genomes (KEGG) analysis describes the pathways involved in genes (Balachandran et al., 2015; Kanehisa et al., 2017).

### Construction of PPI and Screening of Hub Genes

In our study, we used the STRING (Search Tool for the Retrieval of Interacting Genes) online tool, which is designed for predicting protein–protein interactions (PPI), to construct a PPI network of selected genes (Szklarczyk et al., 2015). Using the STRING database, genes with a score ≥0.4 were chosen to build a network model visualized by Cytoscape (v3.8.0) (Smoot et al., 2011). In PPI network, Maximal Clique Centrality (MCC) algorithm was reported to be the most effective method of finding hub nodes. The MCC of each node was calculated by CytoHubba (Chin et al., 2014), a plugin in Cytoscape. In this study, the genes with the top 20 MCC values were considered as hub genes. Subsequently, we used the R package VennDiagram to identify key genes related to tuberculosis pleurisy.

## Results

### Identification of Weighted Gene Co-Expression Modules

**FIGURE 2:**
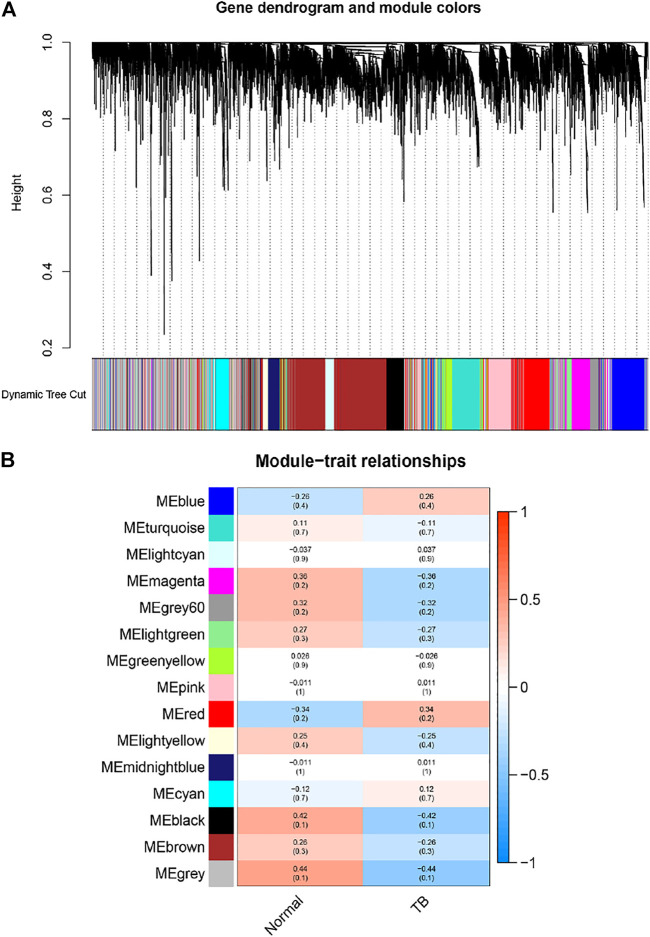
Identification of modules associated with the clinical information in the dataset. **(A)** The Cluster dendrogram of co-expression network modules was ordered by a hierarchical clustering of genes based on the 1-TOM matrix. Each module was assigned different colors. Each module contains genes that belong to the same center in a weighted co-expression network. These genes in module had the same expression profiles. **(B)** Module-trait relationships. Each row corresponds to a color module and column corresponds to a clinical trait (TB and normal). Each cell contains the corresponding correlation and *p*-value. The module with the highest correlation coefficient had the greatest association with tuberculous pleurisy.

In order to find the gene-set most related to the tissue with PET-high, we established a gene weighted co-expression network. Each gene set was treated as a module, and each module was assigned a different color. A total of 15 modules were identified (Figure 2A), and then we drew a module-phenotypic relationship heatmap to evaluate the relationship between modules and clinical phenotypes. The results of the model-phenotypic relationship were shown in Figure 2B, indicating that the grey module in the gene matrix has the highest correlation with the tissue with PET-high.

### Intersection Between DEGs and Co-Expression Modules

**FIGURE 3:**
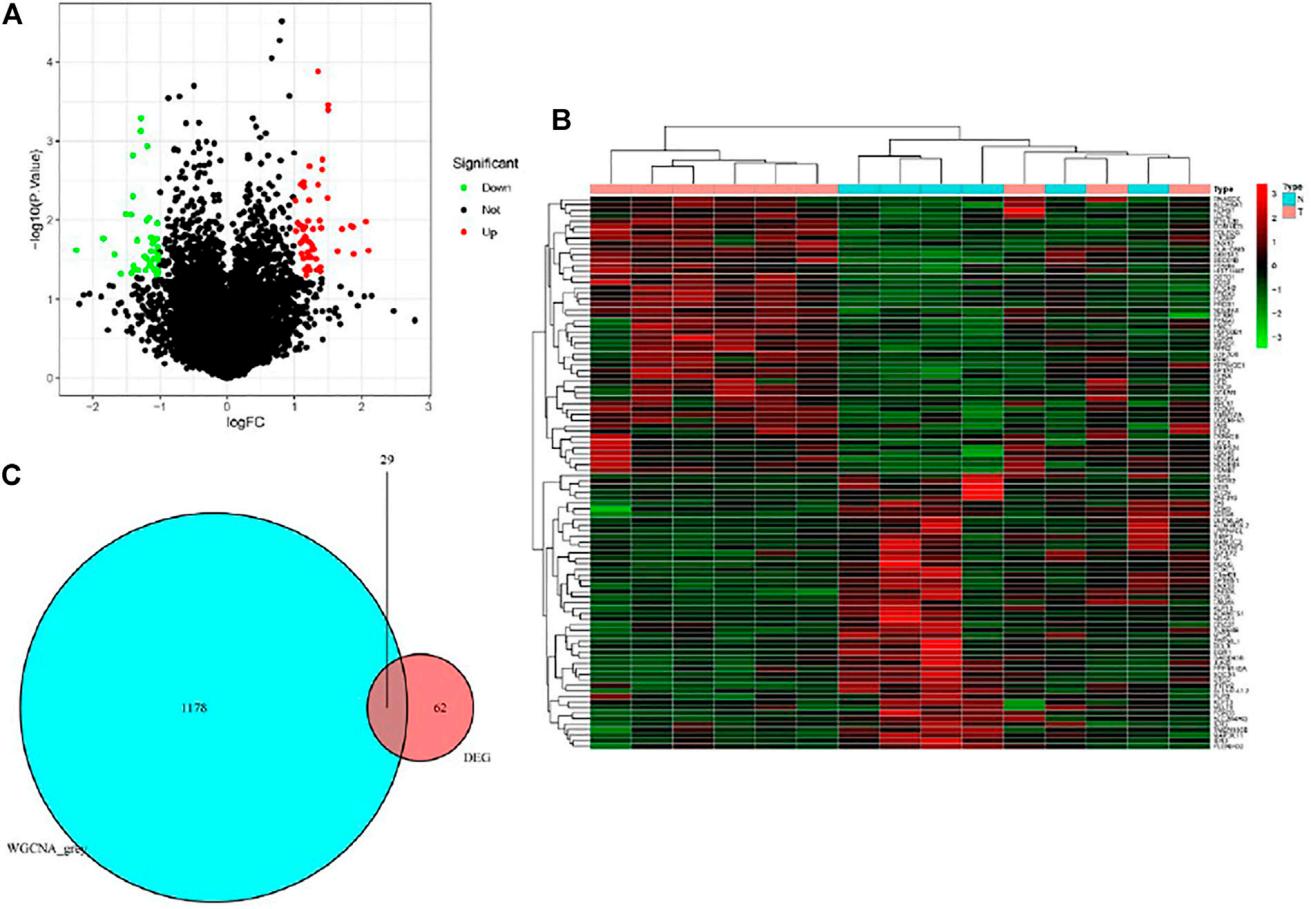
Identification of differentially expressed genes (DEGs) in the datasets with the cut-off criteria of |logFC| ≥ 1.0 and *p* < 0.05. **(A)** Volcano plot of DEGs. **(B)** Heatmap of DEGs. **(C)** The Venn diagram of genes among DEG list and co-expression module. In total, 29 overlapping genes in the intersection of DEG lists and co-expression module.

Based on the cut-off criteria of |logFC| ≥ 1.0 and *p* < 0.05, a total of 62 DEGs in the gene matrix were found to be dysregulated in tissues with PET-high by the limma package (Figures 3A,B). As shown in Figure 3C, 1178 co-expression genes were found in the grey module in gene matrix. In total, the 29 overlapping genes were extracted as key genes in tissues with PET-high (Figure 3C).

### Functional Enrichment Analyses for Selected Genes

**FIGURE 4:**
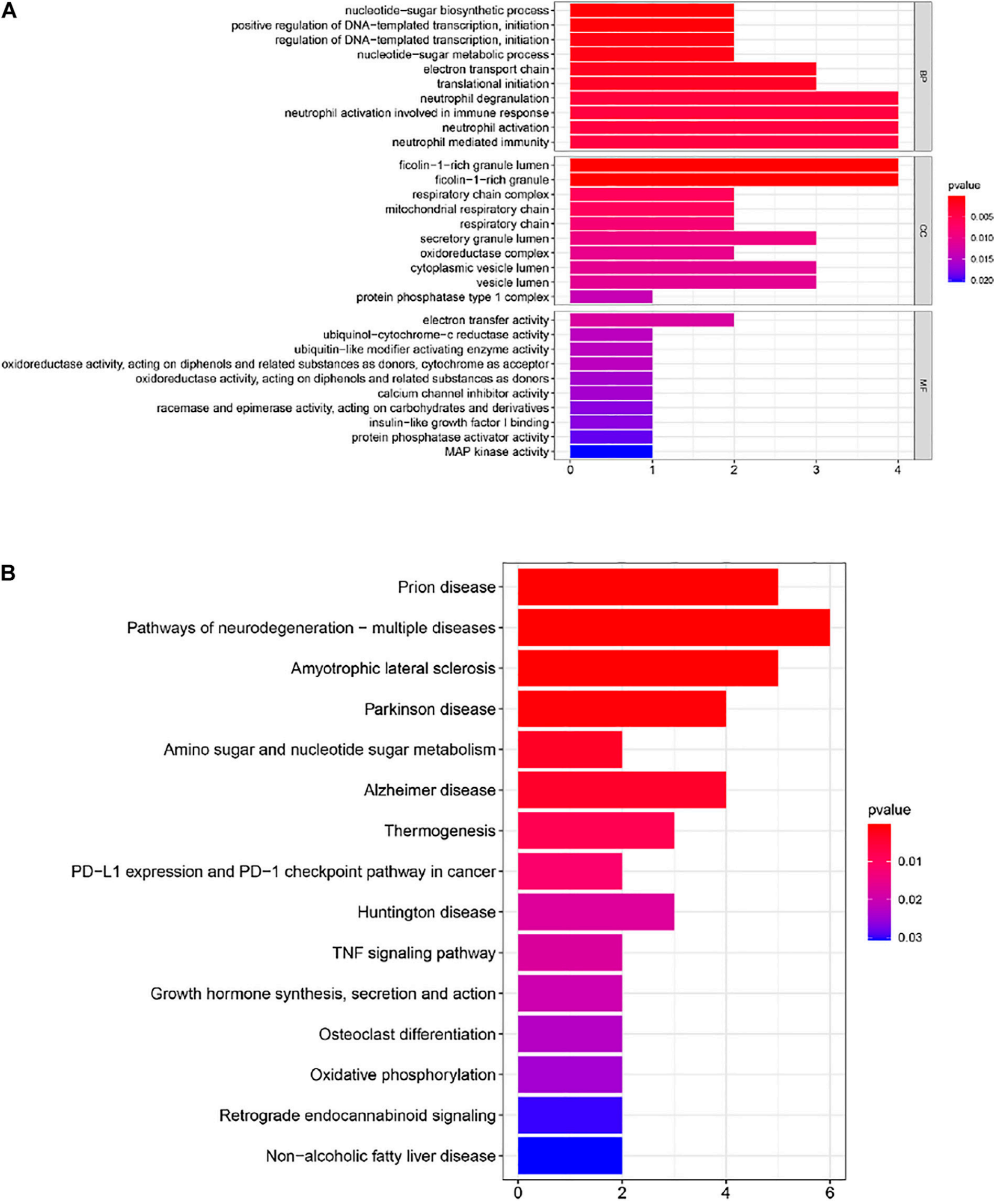
**(A)** Gene Ontology (GO) enrichment analysis for the 29 genes. **(B)** Kyoto Encyclopedia of Genes and Genomes (KEGG) enrichment analysis for 29 genes. The color represents the adjusted *p*-values (BH), and the size of the bars represents the gene number.

In order to further understand the functions and pathways of the co-expression module (Supplementary Table S1), DEGs (Supplementary Table S2) and 29 overlapping genes (Supplementary Table S3), we used clusterProfiler R package for functional enrichment analysis. After screening of GO enrichment analysis, we observed several enriched gene sets shown in Figure 4A; Tables 1, 2. The genes in grey module mainly enriched in cell activation involved in immune response, cellular protein catabolic process and protein domain specific binding. The DEGs mainly enriched in regulation of inflammatory response, regulation of hemopoiesis and negative regulation of immune system process. The 29 overlapping genes mainly enriched in neutrophil degranulation, neutrophil activation involved in immune response and neutrophil activation. After screening of KEGG enrichment analysis, we observed several enriched gene sets shown in Figure 4B; Tables 3, 4. The genes in grey module mainly enriched in Endocytosis, Epstein-Barr virus infection and Human T-cell leukemia virus 1 infection. The DEGs mainly enriched in Complement and coagulation cascades, Proteasome and Arginine and proline metabolism. The 29 overlapping genes mainly enriched in Pathways of neurodegeneration-multiple diseases, Prion disease and Amyotrophic lateral sclerosis.

**TABLE 1:** GO analysis of DEGs.

GO	Category	Description	Count	*p*-value
GO:0050727	GO Biological Processes	regulation of inflammatory response	12	<0.001
GO:1903706	GO Biological Processes	regulation of hemopoiesis	11	<0.001
GO:0002683	GO Biological Processes	negative regulation of immune system process	9	<0.001
GO:0008285	GO Biological Processes	negative regulation of cell proliferation	11	<0.001
GO:0101,002	GO Cellular Components	ficolin-1-rich granule	6	<0.001
GO:0046651	GO Biological Processes	lymphocyte proliferation	7	<0.001
GO:0044106	GO Biological Processes	cellular amine metabolic process	5	<0.001
GO:0030336	GO Biological Processes	negative regulation of cell migration	7	<0.001
GO:0071889	GO Molecular Functions	14-3-3 protein binding	3	<0.001
GO:0090100	GO Biological Processes	positive regulation of transmembrane receptor protein serine/threonine kinase signaling pathway	4	<0.001
GO:0071417	GO Biological Processes	cellular response to organonitrogen compound	8	<0.001
GO:0001667	GO Biological Processes	ameboidal-type cell migration	7	<0.001
GO:0051384	GO Biological Processes	response to glucocorticoid	4	<0.001
GO:0008233	GO Molecular Functions	peptidase activity	8	<0.001
GO:0033013	GO Biological Processes	tetrapyrrole metabolic process	3	<0.001
GO:0055114	GO Biological Processes	oxidation-reduction process	7	<0.001
GO:0070972	GO Biological Processes	protein localization to endoplasmic reticulum	4	<0.001
GO:0000781	GO Cellular Components	chromosome, telomeric region	4	0.002
GO:0005581	GO Cellular Components	collagen trimer	3	0.002
GO:0001227	GO Molecular Functions	DNA-binding transcription repressor activity, RNA polymerase II-specific	5	0.002

**TABLE 2:** KEGG analysis of DEGs.

GO	Category	Description	Count	*p*-value
ko04610	KEGG Pathway	Complement and coagulation cascades	4	<0.001
ko03050	KEGG Pathway	Proteasome	3	<0.001
ko00330	KEGG Pathway	Arginine and proline metabolism	3	<0.001
ko04932	KEGG Pathway	Non-alcoholic fatty liver disease (NAFLD)	4	0.002
ko04657	KEGG Pathway	IL-17 signaling pathway	3	0.003
ko05145	KEGG Pathway	Toxoplasmosis	3	0.005
hsa04068	KEGG Pathway	foxo signaling pathway	3	0.009

**TABLE 3:** GO analysis of WCGNA grey module.

GO	Category	Description	Count	*p*-value
GO:0002263	GO Biological Processes	cell activation involved in immune response	92	<0.001
GO:0044257	GO Biological Processes	cellular protein catabolic process	90	<0.001
GO:0019904	GO Molecular Functions	protein domain specific binding	82	<0.001
GO:0070161	GO Cellular Components	anchoring junction	72	<0.001
GO:0044440	GO Cellular Components	endosomal part	69	<0.001
GO:0072594	GO Biological Processes	establishment of protein localization to organelle	68	<0.001
GO:0005773	GO Cellular Components	vacuole	85	<0.001
GO:0030659	GO Cellular Components	cytoplasmic vesicle membrane	82	<0.001
GO:1903827	GO Biological Processes	regulation of cellular protein localization	63	<0.001
GO:0046700	GO Biological Processes	heterocycle catabolic process	75	<0.001
GO:1901137	GO Biological Processes	carbohydrate derivative biosynthetic process	78	<0.001
GO:1990234	GO Cellular Components	transferase complex	77	<0.001
GO:0003682	GO Molecular Functions	chromatin binding	62	<0.001
GO:0019900	GO Molecular Functions	kinase binding	74	<0.001
GO:0043043	GO Biological Processes	peptide biosynthetic process	72	<0.001
GO:0030135	GO Cellular Components	coated vesicle	40	<0.001
GO:0000139	GO Cellular Components	Golgi membrane	73	<0.001
GO:0051347	GO Biological Processes	positive regulation of transferase activity	67	<0.001
GO:0051129	GO Biological Processes	negative regulation of cellular component organization	73	<0.001
GO:0006091	GO Biological Processes	generation of precursor metabolites and energy	55	<0.001

**TABLE 4:** KEGG analysis of WCGNA grey module.

GO	Category	Description	Count	*p*-value
hsa04144	KEGG Pathway	Endocytosis	38	<0.001
ko05169	KEGG Pathway	Epstein-Barr virus infection	31	<0.001
hsa05166	KEGG Pathway	Human T-cell leukemia virus 1 infection	38	<0.001
ko04962	KEGG Pathway	Vasopressin-regulated water reabsorption	12	<0.001
hsa04931	KEGG Pathway	insulin resistance	19	<0.001
ko04141	KEGG Pathway	Protein processing in endoplasmic reticulum	23	<0.001
hsa04714	KEGG Pathway	thermogenesis	31	<0.001
hsa05200	KEGG Pathway	Pathways in cancer	49	<0.001
hsa05163	KEGG Pathway	human cytomegalovirus infection	27	<0.001
hsa04668	KEGG Pathway	TNF signaling pathway	17	<0.001
hsa05165	KEGG Pathway	human papillomavirus infection	34	<0.001
hsa04066	KEGG Pathway	HIF-1 signaling pathway	17	<0.001
hsa00514	KEGG Pathway	Other types of O-glycan biosynthesis	7	<0.001
ko03015	KEGG Pathway	mRNA surveillance pathway	14	<0.001
hsa04210	KEGG Pathway	Apoptosis	18	<0.001
ko03010	KEGG Pathway	Ribosome	18	<0.001
hsa04621	KEGG Pathway	NOD-like receptor signaling pathway	20	<0.001
hsa05133	KEGG Pathway	Pertussis	12	<0.001
hsa05205	KEGG Pathway	Proteoglycans in cancer	22	<0.001
hsa05146	KEGG Pathway	Amoebiasis	14	<0.001

### PPI Network Construction and Hub Genes Identification

**FIGURE 5:**
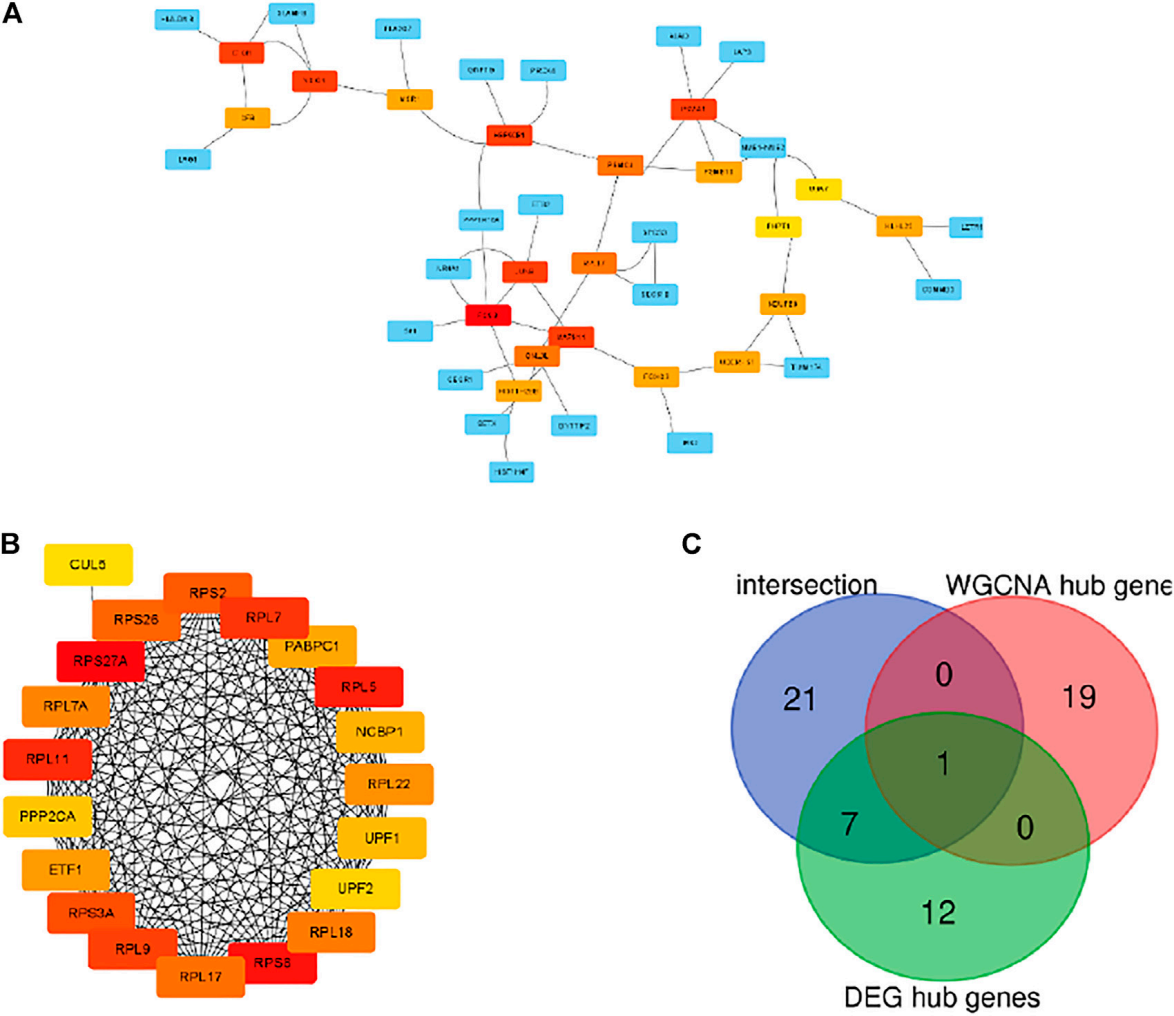
Visualization of the protein-protein interaction (PPI) network and the candidate hub genes. **(A)** PPI network of the hub genes of DEGs list. **(B)** Identification of the hub genes of WGCNA grey module from the PPI network using maximal clique centrality (MCC) algorithm. Edges represent the protein-protein associations. The red nodes represent genes with a high MCC sores, while the yellow node represent genes with a low MCC sore. **(C)** The venn diagram among intersection gene list, WGCNA hub gens and DEG hub genes.

The PPI network of the grey module and DEGs was established by using the STRING database. The hub genes selected from the PPI network using the MCC algorithm of CytoHubba plugin were shown in Figures 5A,B. Then, the top 20 MCC scores genes were identified as hub genes. The intersection among hub genes in grey module, hub genes of DEGs and 29 overlapping genes was used to screen key genes as biomarker (Figure 5C). Finally, one gene named RPL17 was the candidate biomarker, and seven genes included UBA7, NDUFB8, UQCRFS1, JUNB, PSMC4, PHPT1, and MAPK11 can also be the candidate biomarkers.

## Discussion

Tuberculous pleurisy is an uncommon type of tuberculosis in developed countries, but it is more common in developing countries. According to the global report on tuberculosis of the World Health Organization, tuberculous pleurisy accounts for only 3% tuberculosis in developed countries, more than 30% in India and 20% in China. Pleural effusion caused by tuberculous pleurisy can lead to shortness of breath, fever, and other clinical symptoms. However, there are still difficulties in the diagnosis and treatment of tuberculous pleurisy. In this study, through transcriptome sequencing and bioinformatics analysis of surgically resected pleural tissue, we hope to find a biomarker that can be used in the diagnosis and treatment of tuberculous pleurisy. A total of eight possible biomarkers of tuberculous pleurisy were screened (RPL17, UBA7, NDUFB8, UQCRFS1, JUNB, PSMC4, PHPT1, and MAPK11). Through GO analysis and KEGG analysis of these genes, we found that the main functions of these genes were immune response and protein synthesis. In addition, we conducted MCC analysis, and the intersection of the three gene sets was screened out. In the intersection there was only one gene, that is, RPL17. Finally, we determined that RPL17 may be a biomarker of tuberculous pleurisy.

RPL17, also known as ribosomal protein L17, belongs to the L22P family of ribosomal proteins. Ribosome is an important unit involved in protein synthesis, which is composed of 40s subunit, 60s subunit and ribosomal RNA. RPL17 encodes a ribosomal protein in the 60s subunit (Smolock et al., 2012; Zhang et al., 2013). The expression of RPL17 is significantly increased in organs with increased protein synthesis, such as ovaries, bone marrow, and lymph nodes (Wang et al., 2015; Huang et al., 2016). Some studies have pointed out that under the pathological condition, the protein synthesis and metabolism of some organs will be enhanced, which is consistent with our research results (Yuan et al., 2018; Jahejo et al., 2020). However, a study has suggested that RPL17 may act outside the ribosome. A study from the United States showed that RPL17 is an inhibitor of vascular smooth muscle cell growth and can inhibit the formation of the inner lining of the internal carotid artery (Smolock et al., 2012). This suggests that RPL17 is not restricted to acting within the ribosome. It is possible that there is also an extra-ribosomal mechanism of RPL17 in tuberculous pleurisy, which requires further studies to confirm. In tuberculous pleurisy, the enhancement of protein anabolism may be an important mechanism of fibrosis, which is closely related to the diagnosis and treatment of tuberculous pleurisy.

There are several shortcomings in this study. First of all, RPL17 as a diagnostic or therapeutic biomarker requires a large number of blood samples for verification. Secondly, the detailed biomolecular mechanism of RPL17 still needs a lot of experiments to explore. Finally, although we have performed a detailed bioinformatics analysis, some genes that play a key role in the occurrence and development of tuberculous pleurisy may still be missed. We hope to solve the above problems step by step in the follow-up study.

In summary, by integrating WGCNA with differential gene expression analysis, our study generated the significant gene RPL17 that has potential for diagnosis and treatment in tuberculous pleurisy.

## Data Availability

The data presented in the study are deposited in the GEO repository, accession number PRJNA780665.
